# Molecular-Targeted Therapy of Pediatric Acute Myeloid Leukemia

**DOI:** 10.3390/molecules27123911

**Published:** 2022-06-18

**Authors:** Piotr Obszański, Anna Kozłowska, Jakub Wańcowiat, Julia Twardowska, Monika Lejman, Joanna Zawitkowska

**Affiliations:** 1 Student Scientific Society, Department of Pediatric Hematology, Oncology and Transplantology, Medical University of Lublin, Gębali 6, 20-093 Lublin, Poland; obszansky@gmail.com (P.O.); aniakozlowska6@o2.pl (A.K.); 2 Student Scientific Society, Laboratory of Genetic Diagnostics, Medical University of Lublin, Gębali 6, 20-093 Lublin, Poland; kuba707k@gmail.com (J.W.); julka.twardowska@gmail.com (J.T.); 3 Laboratory of Genetic Diagnostics, Medical University of Lublin, Gębali 6, 20-093 Lublin, Poland; monika.lejman@umlub.pl; 4 Department of Pediatric Hematology, Oncology and Transplantology, Medical University of Lublin, Gębali 6, 20-093 Lublin, Poland

**Keywords:** acute myeloid leukemia, target therapies, pediatric AML

## Abstract

Acute myeloid leukemia (AML) accounts for approximately 15–20% of all childhood leukemia cases. The overall survival of children with acute myeloid leukemia does not exceed 82%, and the 5-year event-free survival rates range from 46% to 69%. Such suboptimal outcomes are the result of numerous mutations and epigenetic changes occurring in this disease that adversely affect the susceptibility to treatment and relapse rate. We describe various molecular-targeted therapies that have been developed in recent years to meet these challenges and were or are currently being studied in clinical trials. First introduced in adult AML, novel forms of treatment are slowly beginning to change the therapeutic approach to pediatric AML. Despite promising results of clinical trials investigating new drugs, further clinical studies involving greater numbers of pediatric patients are still needed to improve the outcomes in childhood AML.

## 1. Introduction

Acute myeloid leukemia (AML) is a group of heterogeneous diseases that originate from clones of hematopoietic stem and progenitor cells and myeloid lineage precursors carrying genetic mutations that alter cell proliferation and compromise differentiation. The statistics of the National Institutes of Health (NIH) state that AML is most frequently diagnosed among people aged 65–74 and the percent of new cases in this age group is 25.8%, with 4.3 new cases per 100,000 men and women per year. In comparison, for the group of children, adolescents and young adults under the age of 20, this rate amounted to 0.86 per 100,000 in the years 2014–2018 [1,2]. The occurrence of childhood AML reaches 15–20% of the cases of all acute leukemias in children worldwide, with merely slight differences among the continents or countries. This type of leukemia develops equally among boys and girls of all races younger than 15 years, affecting children with a median age of 6 years [3]. In 2016, the World Health Organization (WHO) proposed an updated classification based on clinical, morphological, immunophenotypical, cytogenetic and molecular characteristics classification [4].

Over a few recent decades, the outcomes of childhood AML treatment have improved. Complete remission (CR) is now achieved in 80% of cases, whereas event-free (EFS) and the overall survival (OS) rates are commonly around 50% and 70%, respectively, due to the high rate of relapse [5]. The therapy of AML is based on intensive multidrug chemotherapy, which might include cladribine, fludarabine, daunorubicin, idarubicin, mitoxantrone, cytarabine and allogeneic hematopoietic stem cell transplantation (allo-HSCT) in poor prognosis patients [6,7,8,9,10,11]. In the current therapeutic protocols, three main prognostic categories are distinguished: favorable, intermediate and adverse. Table 1 shows the AML subtypes divided into the groups of risk. Each group contains types of molecular and cytogenetic abnormalities present in childhood AML, as well as the prevalence for each type.

In Table 2, the outcomes of the pediatric study groups are presented. The survival rates are still unsatisfactory, highlighting the urgent need for the development of new methods of treatment to supplement the existing therapeutic protocols. Most new drugs used in AML therapy are first administered to elderly adults, because they have the worst treatment outcomes among all patients. Hence, before a given medication is introduced to children, it is utilized in adult therapy for a significant amount of time. The best way to predict novel approaches to pediatric AML is to pay close attention to the discoveries occurring in the field of adult AML.

In this review, we present novel molecular-targeted therapy beginning to change the way AML is treated. Due to spatial limitations, our primary goal has been to describe methods that are or have been the focus of clinical research. Therapy for pediatric AML has been adopted from adult AML, the underlying assumption being that both diseases share clinical and biological similarities. Consequently, in our review, extensive use has been made of studies performed on adult populations. However, many differences between pediatric and adult AML exist in terms of gene mutation chromosomal aberrations and differentiation lineage. In stark contrast to adults, most pediatric patients have chromosomal abnormalities, and only 20–26% have a normal karyotype. Children with AML have a lower number of somatic mutations in comparison with adults (5 to 6 vs. 10–13). The incidence of specific mutations varies: *KMT2A*, *RAS*, *KIT* and *WT1* are more common among children. Conversely, *DNMT3A*, *TP53* and *IDH* occur practically only among adults. *FLT3* mutations are evenly distributed across age groups [22]. The only major differences in the molecular targets are *IDH1* and *IDH2* mutations intensively researched in adults but not in children due to their virtual absence in pediatric AML [23].

These differences in no way detract from drawing conclusions about pediatric AML from studies on adult patients. Even in the case of mutations uncommon among adult AML patients, clinical trials of drugs targeting these mutations can be conducted because the adult population is significantly larger than the pediatric AML population. Thus, drugs targeting molecular aberrations occurring in the adult population are successfully utilized in children, as will be shown in the literature presented in our review.

## 2. An Innovative Approach in the Treatment of Pediatric Acute Myeloid Leukemia

### 2.1. Signaling Molecule Inhibitors

#### 2.1.1. FLT3 Inhibitors

**Figure 1:**
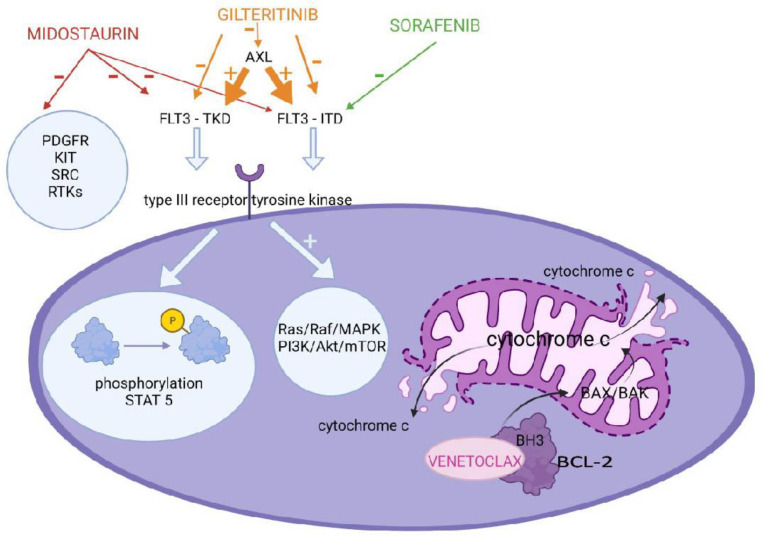
Mechanism of action of the FLT3 and BCL−2 inhibitors. Midostaurin, gilteritinib and sorafenib by inhibiting the mutated type III receptor kinase cause the inhibition of STAT5 phosphorylation and deactivation of the Ras/Raf/MAPK and PI3K/Akt/mTOR pathways, suppressing tumor cell growth. Venetoclax binds to the BH3−binding groove of the BCL−2 protein. This causes the oligomerization and conformational change of BAX and BAK, leading to the formation of pores and mitochondrial outer membrane permeabilization, resulting in tumor cell death.

*FLT3* is one of the most frequently mutated genes in AML, occurring in 20–25% of pediatric patients, and is associated with a poor prognosis. It encodes a transmembrane type III receptor tyrosine kinase (RTK). Activated by a specific ligand, RTK regulates hematopoiesis through the phosphorylation of downstream targets, including STAT5, and activation of critical oncogenic pathways, such as Ras/Raf/MAPK and PI3K/Akt/mTOR (Figure 1).

*FLT3* mutations cause constitutive activation of the tyrosine kinase, causing uninhibited cellular growth. The two major types of *FLT3* mutations are: internal tandem duplication mutations in the juxtamembrane domain (*FLT3*-ITD) and point mutations (the most common is a missense point mutation at the D835) or deletion in the tyrosine kinase domain (*FLT3*-TKD) [24].

Two generations of FLT3 inhibitors have been developed. First-generation inhibitors have multi-kinase activity with a lower specificity for *FLT3* and more off-target effects. Second-generation inhibitors are more specific for FLT3 activity. FLT3 inhibitors are also divided into type I—active against *FLT3*-TKD and *FLT3*-ITD—and type II—active only against ITD [25].

Midostaurin is a first-generation type I FLT3 inhibitor active against PDGFR, KIT, SRC and other RTKs. It received FDA approval in 2017 as the first FLT3 inhibitor in newly diagnosed *FLT3^mut^* adult AML (FDA approval status of the therapies described in this review is presented in Table A1 in Appendix A). Midostuarin is currently evaluated as the treatment in combination with chemotherapy in a phase II trial in pediatric AML (NCT03591510). In the RATIFY trial, the effects of the addition of midostaurin to standard chemotherapy in patients aged 18 to 59 with AML and a *FLT3* mutation were assessed. Median overall survival in the midostaurin group was 74.7 months in comparison to 25.6 in the placebo group with a corresponding hazard ratio for death of 0.78, which amounted to a 22% lower risk of death in the midostaurin group. Median event-free survival (EFS) in the midostaurin group was 8.2 months and 3.0 in the placebo group (outcomes of phase III clinical studies are summarized in Table A2 in Appendix A) [26].

A follow-up study by Voso et al. showed that a 5-year event-free survival rate was significantly higher in the midostaurin group in comparison to the placebo (45% vs. 34%). *NPM1* mutations were a favorable prognostic factor, with the overall survival rate being 69.8% in comparison to 45.7% in *NPM1* wild-type patients [27].

In a phase I/II clinical trial, Zwaan et al. evaluated the safety and efficacy of single-agent oral midostaurin for the first time in children with R/R *FLT3^mut^* AML and *KMT2A*-R ALL. Patients were divided into two dose cohorts: 30 mg/m^2^ and 60 mg/m^2^, with dose-limiting toxicity (DLT) defined as a nonhematological grade 3 or 4 adverse event (AE), drug-related abnormal laboratory value or an AE leading to study discontinuation within 14 days of starting the treatment occurring in the dose-determining set. The lower dose was administered to 7 out of 22 patients (of which, 3 had *FLT3^mut^*), and 15 (6 with *FLT3^mut^*) received the higher dose. No patients in the lower dose group exhibited DLTs. The recommended dose of expansion was set at 60 mg/m^2^ after 77.3% of patients in the studied group experienced grade 3 or 4 adverse events in this dose cohort; however, the study design did not allow dosing beyond 60 mg/m^2^. The efficacy was limited, with an overall survival rate in the *FLT3^mut^* group of 3.7 months and only one patient achieving complete remission but incomplete count recovery at day 14 and being alive 960 days later during the follow-up [28].

Sorafenib, a type II first-generation drug, has been proven to significantly improve the event-free survival and relapse-free survival (RFS) in newly diagnosed AML patients according to the results of the SORAML trial. Patients aged 18–60 years old were divided into two arms—133 received a sorafenib-like placebo with standard chemotherapy, and 134 were given sorafenib with the standard chemotherapy. After a median follow-up of 78 months, it was found that the 5-year EFS in the sorafenib arm was 41% (95% confidence interval) versus 27% in the placebo arm. Among the 159 patients who achieved complete remission, 5-year RFS was 53% in the sorafenib arm and 36% in the placebo arm [29].

Gilteritinib, a type I second-generation agent, is additionally an inhibitor of AXL, a tyrosine kinase responsible for enhancing *FLT3* activation—an important factor in resistance to FLT3 inhibitor therapy. In a phase III ADMIRAL trial, 371 patients aged 18 or older with relapsed or refractory *FLT3*-mutated AML underwent randomization, with 247 of them assigned to receive gilteritinib 120 mg/day, and 124 were administered salvage chemotherapy. The median duration of the follow-up for the overall survival was 17.8 months, and it was significantly longer among patients in the gilteritinib group (9.3 months vs. 5.6 months). The median event-free survival was higher in the gilteritinib group (2.8 vs. 0.7 months), but no statistically significant difference was observed. Complete remission was more frequent in the patients taking gilteritinib (21.1% vs. 10.5%). More patients achieved complete remission with full or partial hematologic recovery in the gilteritinib group (34% vs. 15.3%) [30].

#### 2.1.2. Sub-Analysis of Molecular Therapies for AML with Other Frequent Genetic Mutations

Other genetic mutations frequently occurring in pediatric AML lack the amount of research devoted to *FLT3*. *KMT2A* is a gene encoding zinc-finger protein HRX responsible for the regulation of gene transcription. The rearrangement of *KMT2A* is one of the most frequent in pediatric AML and has been found in 25% of newly diagnosed patients [31]. The results of the AAML0531 phase III clinical trial have proven that Gemtuzumab ozogamicin (GO), an anti-CD33-calicheamicin drug–antibody conjugate, is effective in treating *KMT2A*-mutated pediatric AML. The 5-year EFS was significantly higher in the GO-receiving group (48%) in comparison to the placebo (29%), with both cohorts being administered conventional chemotherapy [32].

The Wilms Tumor gene (*WT1*) encodes a zinc finger motif-containing transcription factor involved in the regulation of cell growth and differentiation. Mutations in this gene occur in 7% of pediatric patients and are associated with a poor prognosis [33]. A novel T-cell bispecific antibody targeting *WT1* has been found to lead cytotoxicity directed against the AML cell lines, leading to the initiation of a phase I trial in adult patients with relapsed or refractory AML (NCT04580121) [34]. Recently, vaccinations with a WT1 recombinant protein have been administered to 5 AML patients (median age 69 years) after standard chemotherapy. In three patients, positive effects were observed consisting of 59-month-long and 18-month-long remission maintenance and complete and sustained MRD clearance. In one case, a complete loss of WT1 expression was observed at relapse [35].

The *RUNX1* gene encodes a transcription factor responsible for hematopoietic stem cell differentiation, and its mutations have been found to occur in 15% of pediatric patients (see Table 1). It has been explored as a potential target with several chemotherapeutics, such as venetoclax or omacetaxine, showing efficacy against AML cells with *RUNX1* mutations in preclinical trials. However, no drug has of yet proven its clinical significance [36].

KIT is a type-III receptor tyrosine kinase contributing to cell signaling, among others, in hematopoietic stem cells. Present in less than 5% of pediatric AML cases, it has been explored as a potential target, with imatinib and dasatinib having shown potential as maintenance therapy to improve the outcomes in adult AML in several clinical trials. Midostaurin has also been investigated as potentially beneficial for patients with *KIT* mutations in the MIDOKIT trial whose results are yet to be published [37].

#### 2.1.3. BCL-2 Inhibitors

The B-cell leukemia/lymphoma 2 (BCL-2) family of proapoptotic and antiapoptotic proteins is crucial to the regulation of the intrinsic mitochondrial pathway of apoptosis. The antiapoptotic group consists of BCL-2, BCL-XL, MCL-1, BCLW and BFL-1 proteins. The pro-apoptotic proteins are divided into effectors: BAK, BAX, BOK and BH3 only proteins further subdivided into sensitizers: BAD, NOXA, BMF, HRK and BIK and activators: BIM, BID and PUMA. Cell death is determined by the interplay between these groups of proteins; however, BH3-only proteins are most important in initiating apoptosis. A few models have been proposed as to how exactly this interplay looks, and all of them share these steps—oligomerization and the conformational change of BAX and BAK, leading to BAX/BAK pore formation and mitochondrial outer membrane permeabilization, resulting in cytochrome c release into the cytoplasm (Figure 1) [38]. The disruption of the equilibrium between member proteins in favor of the proapoptotic group is an important factor in neoplasm growth. Various mechanisms have been shown to account for this including overexpression of antiapoptotic BCL-2 members (e.g., BCL-2), loss or downregulation of the proapoptotic BCL-2 members, impairment of BH3-only proteins due to the p53 deficiency and epigenetic aberration, such as the silencing of BIM and PUMA through promoter hypermethylation. The discovery of BCL-2 proteins has prompted the development of novel therapeutic agents that inhibit the activity of the antiapoptotic, pro-survival BCL-2 member proteins. After the first drugs designed in this class (navitoclax and obatoclax) whose mechanism of action was to inhibit all antiapoptotic BCL-2 proteins, were shown to cause significant adverse effects such as thrombocytopenia due to BCL-XL inhibition, attention was given to developing more selective agents. Venetoclax is a BH3 mimetic that binds with high affinity to the BH3-binding groove of the BCL-2 protein [39].

Wei et al. assessed the safety and efficacy of venetoclax in combination with low-dose cytarabine in patients with previously untreated AML aged 60–93 years (median age 74 years). All patients were observed for at least 6 months. Of the group numbering 82 patients at the start of the study, the CR/CRi (CR—complete remission, CRi—complete remission with incomplete blood count recovery) rate was 54%, of which CR was achieved in 28% and CRi in 26% of patients. The median duration of remission was 8.1 months, and the median overall survival was 10.1 months. The most common grade 3 or 4 adverse events were neutropenia (42%), thrombocytopenia (38%), febrile neutropenia (27%) and anemia (27%). AML progression was observed in seven patients (9%) [40].

In a follow-up phase III trial—NCT03069352, 210 previously untreated patients aged 36–93 years were evaluated (median age 76 years) after randomization to low-dose cytarabine with venetoclax (142 patients) or the placebo (68 patients). The median overall survival was 8.4 months in the venetoclax arm and 4.1 months in the placebo arm after prolonging the follow-up time by 6 months after the study cut-off date. The CR/CRi rate and median event-free survival time were significantly higher in the venetoclax arm (48% and 4.7 months) in comparison to the placebo (13% and 2.0 months). Adverse event (AE) occurrence was comparable between both arms, with 99% of all patients experiencing at least one adverse event, with tumor lysis syndrome being the only exception (eight cases total in the venetoclax arm). Serious adverse events, the most common being febrile neutropenia (16% vs. 18%), pneumonia (13% vs. 10%) and sepsis (6% in both arms), occurred in 66% of patients in the venetoclax arm and 62% of the placebo arm. Adverse events leading to death and a 30-day mortality rate were similar in both arms, respectively, at 23% vs. 21% and 13% vs. 16% [41].

Encouraging results emerged from the retrospectively collected data from Israel where 40 patients aged 21–82 years (median age 67 years) with relapsed/refractory AML were treated between 2016 and 2019. Receiving at least one aggressive chemotherapy regimen was an eligibility criterion. Prior allogeneic hematopoietic cell transplantation did not lead to exclusion from the analysis. The median time from diagnosis to the commencement of venetoclax therapy was 6 months, and the drug was administered after a median of two prior lines of treatment (not including HCT). No instances of tumor lysis syndrome were observed. Eleven patients (27.5%) did not survive more than 2 months, and the most important causes of death were disease progression (seven patients, 64%) or infection (two patients, 18%). Survival longer than 2 months was noted in 29 cases, of which 22 (76%) achieved neutrophil recovery and 17 (59%) also platelet count recovery. CR/CRi was confirmed by bone marrow examination in 15 patients (52% of the >2 months survival group and 68% of those who achieved at least neutrophil recovery) [42].

The efficacy and safety of the combined treatment with venetoclax and azacitidine in pediatric patients with relapsed/refractory AML were reported in a retrospective analysis of the patients treated by Winters et al. The total number of patients included in the report was eight; six had AML and two MDS. Of the six AML patients, aged 2–20 years, three patients achieved CR and were reported later as minimal residual disease (MRD)-negative after a median follow-up of 6.4 months since the commencement of venetoclax therapy. One patient received venetoclax and azacitidine in combination with gilteritinib due to *FLT3* internal tandem duplication-positive AML that proved refractory to two cycles of conventional chemotherapy and became severely hypoplastic (MLFS), and in that state, HSCT was performed and was later reported as MRD-negative. Of the two patients who did not respond to therapy, one had congenital *KMT2A*-rearranged ALL with a lineage switch after CAR-T therapy and another AML as a second malignancy in the setting of a DNA damage repair defect (Bloom Syndrome). There were no grade 5 AEs, and of grade 4 AEs, the most common were neutropenia and thrombocytopenia (experienced by all patients) and febrile neutropenia (three cases) [43].

A phase I trial investigating the safety and pharmacokinetics of venetoclax monotherapy, followed by the addition of chemotherapy in pediatric and young adult patients with relapsed/refractory malignancies (ALL, AML, NHL, neuroblastoma and other tumors), is currently in the recruitment stage (NCT03236857) [44].

### 2.2. Epigenetic Modifier Inhibitors

#### 2.2.1. Hypomethylating Agents

**Figure 2:**
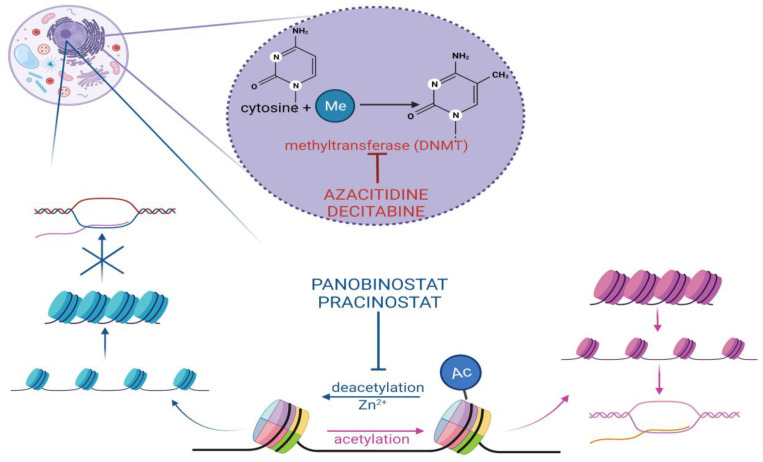
Mechanisms of action of epigenetic modifiers. Azacitidine and decitabine by inhibiting DNA methyltransferase reverse the aberrant hypermethylation of genes, which is a crucial epigenetic component of leukemic transformation. Pracinostat and panobinostat inhibit histone deacetylation, causing the re-expression of the genes involved in cell differentiation.

Cytidine analogs azacitidine (AZA) and decitabine (DAC) inhibit DNA methyltransferase (DNMT), a crucial enzyme in the process of DNA methylation. DNMT catalyzes the addition of methyl groups to nucleotide cytosine (Figure 2). It is one of the forms of the epigenetic regulation of the gene expression and is responsible under physiological conditions for cellular differentiation and the suppression of retroviral elements. A distinct set of aberrantly methylated and silenced genes, likely involved in an epigenetic pathway in the leukemic transformation process, has been shown to account for the molecular diversity observed in AML [45]. Hypomethylating agents (HMAs) allow for the re-expression of important genes involved in cell differentiation and cell cycle control and have proved to be an effective treatment method of AML, especially in combination with venetoclax [46].

There has been some debate regarding the exact antitumor mechanism of these drugs. HMAs currently form the backbone of the non-intensive treatment of AML, especially in those patients with adverse genetics such as monosomal karyotypes, often with losses on chromosomes 7, 5 or 17. In a study by Greve et al., RNA-seq was used to compare the effects of DAC with cytarabine (AraC), which is a cytidine analog without hypomethylating properties. Both substances were used in cell lines and murine models with monosomal karyotype AML. Azacitidine and decitabine were found to reverse the compounding hypermethylation and gene silencing of hemizygous tumor suppressor genes (TSG). For this purpose, AML cell lines: UCSD-AML1 (AML1) and ELF-153 (ELF) were treated with DAC at doses that did not induce cell death. The assay for Transposase Accessible Chromatin (ATAC)- and RNA-sequencing of DAC-treated cells disclosed a massive 254 increase in accessible chromatin sites, leading to several thousands of significantly 255 differentially expressed protein-coding transcripts (DETs) in AML1 and ELF, respectively (upregulated: 1704/1205; downregulated: 2,571,879/661). Monosomal karyotype AML murine models treated with DAC, AraC or the vehicle confirmed the superior antitumor activity of the HMAs in this type of leukemia—the median survival was prolonged by 41 days by DAC in comparison to the vehicle and by 18 days by AraC in comparison to the vehicle [47].

Novel oral azacitidne was assessed for safety and efficacy as a maintenance therapy in patients with AML who were in their first remission after intensive chemotherapy in a phase III trial by Wei et al. Patients (*n* = 472) aged 55 to 86 years old (median age 68) underwent randomization into the azacitidine arm (238 patients) and the placebo arm (234 patients). The median overall survival was significantly longer in the azacitidine arm (24.7 months) than in the placebo arm (14.8 months). The median relapse-free survival was also significantly longer in the azacitidine group (10.2 months) in comparison with the placebo group (4.8 months). Grade 3 or 4 adverse events were observed in 72% of patients in the azacitidine arm and 63% of the patients in the placebo arm, the most common being neutropenia (42% in the azacitidne group vs. 24% in the placebo group), thrombocytopenia (respectively 22% vs. 21%) and anemia (14% vs. 13%) [48].

Decitabine in combination with fludarabine, cytarabine and G-CSF (FLAG chemotherapy) and vorinostat (a histone deacetylase inhibitor) have been investigated in pediatric patients with R/R AML in a phase I study by Pommert et al. Thirty-seven patients aged 1–20 (median age 8.4) were enrolled and, eventually, 35 evaluated, with two patients removed from the study, one due to rapidly progressive disease and the other due to death from rapidly progressive disease prior to receiving 80% of the planned therapy. The most common grade 3 to 4 adverse events observed were hypokalemia (35%), anorexia (17%), elevated AST (17%) and hypoxia (17%). Thirty-five out of the thirty-seven enrolled patients were evaluable for a response; two patients were excluded from the analysis due to death from seizure and asystole secondary to a presumed intracranial event after receiving one dose of vorinostat/decitabine (*n* = 1) and a hypocellular marrow (*n* = 1). CR/CRi was achieved in 54% of patients (*n* = 19; 16 CR, 3 Cri), of which 90% (*n* = 17) of the responding patients achieved MRD negativity (<0.1%) by centralized flow cytometry. Patients who achieved CR MRD- (*n* = 17) had a 2-year OS of 75.6% versus 17.9% for those with residual disease (*n* = 18) [49].

Cheng et al. presented three cases of pediatric patients (aged 10, 11 and 16 years old) with AML with monosomy 5/del(5q) treated with combined low-dose cytarabine and mitoxantrone concurrently with decitabine and G-CSF for remission induction. The first patient was a 10-year-old boy with primary AML with a t(7;21)(p22;q22) translocation and mutations in *NRAS* and *WT1*. He tolerated the treatment well and achieved CR. Finally, he underwent matched unrelated donor HCT and was alive and free of disease 3.6 years after the transplant. The second patient, an 11-year-old girl with prior treatment for B-cell ALL, according to the CCLG-ALL-2008 protocol and Li-Fraumeni syndrome, had AML with a *TP53* mutation. She achieved CR and, later on, a haploidentical HCT from her father. The patient remains in remission at 3.2 years after the transplant. The third reported case was of a 16-year-old girl with primary AML with mutations in *SF3B1* and *EZH2*. She also achieved CR before proceeding to haploidentical HCT from her father and remains in remission 3.0 years after the transplant [50].

#### 2.2.2. Histone Deacetylase Inhibitors

Forms of epigenetic regulation have recently been found to play a tremendous role in the process of leukemogenesis. This discovery has led to the development of new forms of therapy in AML, among which are histone deacetylase inhibitors (HDACIs). The acetylation and deacetylation of the histone tail constitute one of the best-characterized aspects of epigenetic regulation (Figure 2). It consists of the acetylation or deacetylation of lysine residues present mainly in the N-terminal region of histone tails. Neutralization of the positive charge in lysine caused by acetylation leads to weakening of the electrostatic interaction between histone and negatively charged DNA, opening the chromatin structure. Thus, the binding of transcription factors resulting in gene expression is enabled. On the other hand, histone deacetylation condenses the chromatin structure, decreasing or inhibiting gene transcription. Processes crucial to cell life, such as replication, chromatin packing, DNA repair or apoptosis, are regulated through the acetylation or deacetylation of histones. HDAC are divided into four classes, depending on either the Zn2+ or NAD+ cofactors. Classes I, II and IV have a zinc cofactor and include the following HDAC members (HDAC 1–11). Class III has a NAD+ cofactor and consists of the sirtuin (SIRT)1–7 sirtuin protein family. Histone deacetylase (HDAC) genes have not been found to be mutated in AML. However, HDACs are recruited to specific gene promoters by abnormal oncogenic fusion proteins occurring in this disease, such as PML-RARA, PLZF-RARA or AML1-ETO. This aberrant silencing of genes contributes to leukemogenesis through cell differentiation arrest [51,52].

Panobinostat is a hydroxamic acid-based inhibitor of mainly HDAC 1, 2, 3 and 6, especially implicated in cancer development. DeAngelo et al. examined 46 patients aged 18–65 years (median age 55 years) with primary (*n* = 36) and secondary (*n* = 10) AML treated with panobinostat combined with idarubicin. Twenty patients (43.5%) achieved CR, and eight patients (17.4%) achieved Cri; thus, the overall response rate (CR or CRi) was 60.9%. The 1-year EFS was 78.3%. Grade 3 or above AEs were experienced by 19 patients (41.3%), of which the most common were: febrile neutropenia (*n* = 10), infection (*n* = 5) and cardiac disorders (*n* = 5). One treatment-related death was observed due to sepsis [53].

Pracinostat, a potent oral pan-HDAC inhibitor, has recently been assessed in combination with azacitidine as therapy for patients with AML aged 65 years old or more and ineligible for standard chemotherapy. Out of 50 patients enrolled in a phase III single-arm study, 21 achieved CR (42%) and 2 achieved CRi (4%). Most common amongst treatment-related grade 3 or 4 AEs, which were experienced by 43 patients (86%), were: infections (26 patients, 52%), thrombocytopenia (23 patients, 46%), febrile neutropenia (22 patients, 44%), neutropenia (19 patients, 38%) and anemia (15 patients, 30%). The median OS was 19.1 months, and the 1-year OS rate was 62% [54].

In a recent meta-analysis, the safety and efficacy of AML with HMAs alone or in combination with HDAC inhibitors were compared. After analyzing seven studies comprising 922 patients (458 patients treated with HMA alone and 464 with HMA and HDACi), no significant differences in the complete remission rates, hematologic improvement, overall response rate, overall survival and toxicities between the HMA alone treatment and HMA with HDAC inhibitor regimens were observed [55].

Panobinostat has also been investigated in a phase I trial as a potential new therapy for pediatric patients with relapsed and refractory AML. Of seventeen enrolled patients, including seven with previous HCT, eight (47%) achieved complete morphologic remission (six patients in this group were also negative for MRD), one achieved a partial response and eight were non-responders. Amongst grade 3 or 4 adverse effects, the most important were 14 episodes of febrile neutropenia and 9 infections, including one case of sepsis [56].

### 2.3. Immunotherapy

#### 2.3.1. Drug–Antibody Conjugates

Antibody–drug conjugates (ADCs) have been the most successful form of immunotherapy to date in treating pediatric acute myeloid leukemia. ADCs consist of a monoclonal antibody joined to a cytotoxic agent by a cleavable linker. This form of therapy is hopefully the way forward in enhancing the antitumor potential of the known chemotherapeutics while significantly reducing their side effects. These benefits stem from the capacity of the monoclonal antibody to bind itself to a receptor found preferably only on the surface of the tumor cell, which causes the internalization of the ADC in the targeted cell. The linker is then cleaved either through an enzymatic reaction or hydrolyzation, and the cytotoxic payload is released. Two types of agents are used as the cytotoxic component of the ADC–microtubule inhibitors and DNA-damaging drugs [57]. Examples of DNA-damaging drugs and their exact mechanisms include: calicheamicin—double-stranded DNA breakage, duocarmycin—DNA alkylation and pyrrolobenzodiazepine dimers—crosslinking with DNA [58]. Gemtuzumab ozogamicin (GO) is the only antibody–drug conjugate approved by both the Food and Drug Administration (in 2020) and European Medicines Agency in newly diagnosed and refractory CD33-positive AML for patients aged 1 month or older. A combination of anti-CD33 IG4 and calicheamicin as the cytotoxic agent GO has been shown to improve event-free survival, though not the overall survival, when used with the standard chemotherapy [59]. In the ALFA-0701 trial, assessing the safety and efficacy of GO, patients aged 50–70 years old with de novo AML were given Gemtuzumab ozogamicin in addition to the standard induction chemotherapy; the control group consisted of patients given only the standard chemotherapy. No statistical differences in the response rate or overall survival were observed between both groups. Event-free survival was significantly longer in the GO arm (median 17.3 months) in comparison with the non-GO arm (median 9.5 months). Regarding adverse effects, hemorrhage, veno-occlusive disease and the number of patients who discontinued therapy because of adverse effects were higher in the study group [60]. In the COG AAML0531 trial, a group of 1022 patients (aged 1 month to 29.99 years) with previously untreated primary AML were divided into two study arms: standard therapy only (non-GO) and standard therapy with GO (each dose 3 mg/m^2^) and evaluated after a median follow-up period of 4.1 years. The relapse risk was found to be significantly lower in the GO arm (32.8% ± 4.6%) vs. the non-GO arm (41.3% ± 4.9%); however, the overall survival was similar in both groups. Grades 3–5 adverse events occurred with comparable frequencies in both study arms, and the prolonged neutrophil recovery time (>59 days) was longer in the GO arm (12.0% vs. 6.3%) [61].

A novel antibody–drug conjugate Camidanlumab tesirine targeting CD25 underwent a phase I clinical trial in patients with relapsed/refractory ALL/AML. CD25 or T-cell activation antigen is an important component in immune function regulation; physiologically found only on activated T and B cells and regulatory T cells (Tregs), its cell surface expression on AML blast cells is linked to the failure of induction therapy, increased risk of relapse and short overall survival. Patients with AML were divided into two groups—one receiving Camidanlumab tesirine every three weeks and further subdivided into eight dose cohorts (3–92 μg/kg) consisting of 26 patients, while group two had the agent administered every week and was subdivided into two dose groups of 30 μg/kg and 37.5 μg/kg. One dose-limiting toxicity was observed during the study—a possibly drug-related grade 3 maculopapular rash in the 30 μg/kg group. Treatment-emergent serious adverse events were experienced by 23 patients (65.7%), of which 11 patients (31.4%) had fatal outcomes; these were disease progression (nine patients), cardiac arrest (one patient) and pneumonia (one patient). No serious adverse effects were considered related to the study drug. Three cases of maculopapular rash and one case each of abdominal pain, decreased appetite and failure to thrive were considered at least possibly related to the study drug among the serious adverse events. There were no dose reductions or treatment interruptions [62]. Targets for potential ADC, often expressed on the surface of AML cells, currently undergoing clinical trials include CD123 and CLL-1 [63,64].

#### 2.3.2. Chimeric Antigen Receptor T Cells

One of the forms of immunotherapy based on genetic engineering is chimeric antigen receptor (CAR) T cells. T cells are collected from the patients’ blood, and a previously modified gene encoding the new receptor is transferred to their DNA through various means, for example, viral vectors or mRNA electroporation. The product of this gene is a new receptor that binds to the cancer-specific antigen. T cells are then expanded ex vivo and infused into the patient [65]. Hence, the primary function of CARs is to target and initiate T-cell activation. Chimeric antigen receptors are comprised of an antigen recognition domain, typically a single-chain variable fragment (scFv) of an antibody, costimulatory domains, transmembrane and hinge components and an activation domain gleaned from the CD3ζ part of the T-cell receptor [66]. Four generations of CAR T cells have been developed and distinguished according to their costimulatory molecules. The first generation consists of the scFv region and the CD3ζ chain and forms the basis for subsequent generations. The second generation possesses an additional costimulatory domain, and third generation CARs possess two. Fourth-generation CAR-T cells are additionally engineered to secrete a transgenic cytokine upon CAR signaling in the targeted tumor tissue [67]. Thus, the redirection of immune reactivity towards the desired antigen is not the only goal of engineering CARs. T-cell proliferation, endurance and the measure of activation within tumor microenvironment are affected by CARs, which leads to a significant alteration in the safety and efficacy of cancer-targeted T cells. CARS targeting CD19 and CD22 have proven to be an effective treatment for B-acute lymphoblastic leukemia and diffuse large B-cell lymphoma [68]. The spectacular results achieved in these diseases are yet to be replicated in AML, where considerable heterogeneity hampers the search for adequate tumor-specific antigens. In the search for the greater persistence of CAR engineered cells and due to significant myeloablative effects of standard CAR T-cell therapy, other types of cells such as NK or cytokine-induced killer cells have been proposed as potential candidates for fusion with CARs. In a study by Rotiroi et al., cytokine-induced killer cells engineered with CD33, CAR were shown to efficiently target drug-resistant AML cells in patient-derived xenografts (PDX) in mice, delaying leukemia progression following the standard chemotherapy treatment [69].

A phase I clinical trial by Tang et al. showed that no significant adverse effects followed the administration of CD33-CAR NK-92 cells to three patients, of which one was a 14-year-old girl, with AML1-ETO(+), C-KIT(+) and M4 AML (intermediate risk) in doses up to 5 × 10^9^ per patient. The other two patients were a 24-year-old man diagnosed with M4 AML containing chromosomal abnormality t(3;16) and a 49-year-old woman diagnosed with CD33+ M4 AML with normal karyotype and multiple mutations, including *NRAS*(+), *NPM1*(+), *DNMT3A*(+) and *TET2*(+) [70].

NKG2D ligands (such as MICA, MICB and ULBP) are a promising target in AML therapy due to their relative absence from healthy cells. In a phase I first-in-human study by Baumaister et al., it was observed that no severe adverse effects (infusional toxicity, cytokine release syndrome, significant autoimmune reactions or death) were observed after administering median transduced T-cell doses ranging from 7.38 × 10^5^ to 2.45 × 10^7^ to seven patients with AML. Unfortunately, the therapeutic effect was slight, with the median overall survival rate being 4.7 months; all patients subsequently received other forms of therapy, the earliest at 28 days after infusion and 6 months at the latest [71].

In a preclinical study following the trial by Driouk et al., NKG2D-CAR T cells were found to demonstrate a high degree of cytotoxic activity against unsorted primary AML blasts in vitro despite a low level of ligand expression ascertained by NKG2D-Fc in comparison to the empty control T cells. Furthermore, low-level NKG2D ligand AML cells exhibited an upregulation of the ligands after being treated with HDAC inhibitor valproic acid, which increased the lytic effect of NKG2D-CAR Ts on the tumor cells [72].

CLL-1, C-type lectin-like molecule-1, a type II transmembrane glycoprotein, is another promising target highly expressed on the surface of AML cells in 87% of patients but not on normal hematopoietic stem cells. Additionally, cytokine exposure can enhance the antitumor capacities of CAR T cells. In a study by Ataca Atilla et al., T cells co-expressing anti-CLL-1 CAR and IL-15 were tested in AML xenograft mouse models. Sublethal-irradiated mice injected first with CLL-1-positive AML cells were then administered two types of CD28z-CD8 CLL-1 CAR T cells: the study group transduced with a human gene encoding IL-15 with an embedded iC9 safety switch and the control group without it. All mice injected with IL-15 co-expressing CAR T cells developed a rapidly progressive shock-like syndrome leading to death associated with rapid tumor destruction, with tumor burden being one log lower than in mice administered with T cells without the IL-15 gene. Elevated levels of TNFα, IL15 and IL2 in comparison to the control group were found to be associated with organ damage in mice and led to applying TNFα-blocking antibodies and CID (activator of the iC9 embedded suicide switch) to the study group. Only when administered sequentially was the antitumor activity maintained and toxicity decreased with mice treated with CLL-1 CAR +IL15 + anti-TNF + CID surviving over 40 days in comparison to the 5-day survival of mice treated with CLL-1 CAR + IL15 and the tumor cell count remaining below 10^1^/100μLat day 30 after injection, whereas, in the non-transduced group, it was above 10^5^/100μL [73].

Zhang et al. reported a morphological, immunophenotypic and molecular complete remission for over 10 months after administering a single dose of 5.8 × 10^7^ anti-CLL1 CAR-T cells ~1.9 × 10^6^/kg to a 10-year-old girl with secondary AML after high-risk B-ALL [74]. The AML-specific targets that are being evaluated in phase I or II clinical trials are CD7, CD33, CD38 CD123, FLT3, CD70 and CLL-1 [75,76,77,78,79,80,81]. Currently, numerous clinical trials of the aforementioned molecular-targeted methods of pediatric AML treatment are being conducted worldwide.

### 2.4. Future Directions

**Figure 3:**
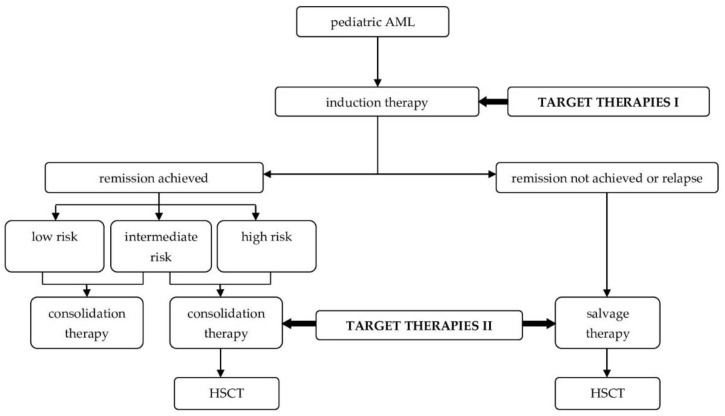
Place of novel AML therapies in the treatment algorithm. Target Therapies I include: Gemtuzumab ozogamicin, midostaurin, sorafenib, venetoclax, azacitidine, decitabine, panobinostat and CAR-T. Target Therapies II include: midostaurin, gilteritinib, venetoclax, azacitidine, decitabine, pracinostat, panobinostat and Camidanlumab tesrine.

Figure 3 presents the proposed future treatment algorithm of AML after the inclusion of novel methods into clinical practice.

#### 2.4.1. Bispecific and Trispecific Antibodies

Bispecific T-engaging antibodies (BiTEs) are small molecules composed of two different antigen–recognition domains derived from variable regions of monoclonal antibody engineered to engage two antigens—CD3 on the surface of T cells and a tumor-associated antigen (TAA) [82]. After being bound both to the CD3 and TAA, the T-cell receptor is activated, causing a cytotoxic response against the tumor cell and thus reducing the off-target toxicity, as the activation will only take place in the presence of targeted cancer cells. Several variants of this design exist—DARTs, BiKEs and TRiKEs. Dual affinity retargets antibodies (DARTs) also consist of two scFVs—CD3 and TAA but with an additional disulfide bridge improving stabilization. Bispecific killer engager antibodies (BiKEs) bind to CD16 instead of CD3, activating natural killer cells, and trispecific killer engager antibodies (TriKEs) possess an IL-15 crosslinker, enhancing the NK cell response [83]. AMG 330, a CD33/CD3-bispecific antibody added to PBMC samples from AML patients to create short-term (3–6 days) cell cultures, has been shown to effectively eliminate CD33+ AML blasts and cause an expansion of residual autologous T cells. Representative flow cytometry has revealed the elevation of the parameter’s indicative of T-cell activation (CD25 and CD69), cytotoxic activity (granzyme B and CD107) or cytokine production (IL2 and IFNγ) in the cell cultures. No influence of the initial T-cell frequency within the PBMCs ranging from 0.16 to 14.30% on the assessed levels of T-cell responsiveness was noted [84]. Hutmacher et al. developed a new anti-CD123 antibody called H9 and compared its antileukemic activity against a previously engineered antibody, CSL362, after reformatting both antibodies into BiTEs by fusing them with an anti-CD3 scFV OKT3. Concentration-dependent AML killing was observed in an in vitro assay, with patient’s derived AML blasts and autologous T cells acting as effectors and an E:T ratio of 10:1. BiTE (CSL362/OKT3) was approximately 10times more potent than BiTE (H9/OKT3), with an IC50 value of 4 ng/mL (corresponding to 7.3 pM) [85]. Two novel anti-FLT3 BiTEs were developed and tested in vitro, in vivo and ex vivo for T-cell-dependent cellular toxicity (TDCC) against FLT3-positive cell lines by Brauchle et al. The two agents evaluated were: an experimental FLT3 BiTE molecule consisting of an anti-CD3 scFv and an anti-FLT3 scFv and a FLT3 half-life extended (HLE) BiTE molecule (AMG 427) consisting of an anti-CD3 scFv fused to an Fc moiety and a unique anti-FLT3 scFv, allowing for an extended serum half-life. The experimental FLT3 BiTE molecule required administration by continuous intravenous infusion to maintain an active concentration, because due to its size, it was quickly cleared by glomerular filtration. TDCC was comparably induced against five FLT3 protein-expressing cell lines with a single-digit picomolar potency by both molecules. Probably because of a high E:T ratio, no apparent relationship between the FLT3 expression level and potency was observed. An increase of the T-cell activation markers CD69 and CD25 and secretion of T-cell-derived effector cytokines IFNg and TNFa in the presence of FLT3 protein-expressing cells, but not in the presence of FLT3 protein-negative cells, were the concomitant effects of TDCC.AMG 427 was evaluated in two mouse xenograft models injected with either EOL-1 or MOLM-13 AML cells and with in vitro expanded human CD3þ T cells, in EOL-1 with a median survival of 36 and 37 days and in all dose group treatments with AMG 427 prolonged survival, with 17 out of 30 animals surviving until the study ended on day 108. AMG 427 significantly extended the survival in comparison with the vehicle (*n* = ¼ 10, *p* < 0.001). All mice in the control groups died within 20 days after injection of the more aggressive MOLM-13 AML cells, with the median survival being 18 days [86].

#### 2.4.2. Paradigm Shift

Until recently, novel treatment strategies for pediatric AML have been translated in the field of adult AML research on the assumption that both diseases are essentially similar. However, recent advances in molecular profiling have shown that aspects crucial for the outcomes such as resistance to induction chemotherapy are associated with age-specific factors such as the interactions of leukemic cells with the bone marrow matrix, age of cells undergoing leukemogenesis and types of mutations in epigenetic modulators. Using murine models implanted with myeloid progenitor cells extracted from differently aged mice to represent infant/prenatal, child, young adult and middle/old age leukemia origins, Chaudhury et al. demonstrated that the age of the cell of origin determines the disease latency, lineage phenotype and bone marrow niche. These discoveries will pave the way for the development of future forms of therapy that will be age-determined and specific to children. Further molecular profiling will identify age-specific targetable vulnerabilities [87].

## 3. Conclusions

The desire to improve the prognosis in pediatric AML, though it has improved in recent years but not satisfactorily, has led to the introduction of various novel methods of treatment. These methods targeting genetic mutations, processes leading to epigenetic aberrations and tumor cell surface markers have led to therapeutic success in a few trials and cases, but further clinical studies involving greater numbers of pediatric patients than the ones conducted to date are needed to make molecular-targeted therapies a solid outcome-altering addition to the standard chemotherapy regimens. The number of currently active or recruiting clinical trials, especially in the field of immunotherapy, BCL-2 inhibitors and hypomethylating agents, are indicative of a surge of interest in these forms of treatment that will hopefully lead to even better outcomes in pediatric AML in the near future.

## Figures and Tables

**Table 1:** Risk stratification based on molecular and cytogenetic abnormalities occurring in AML.

Risk	Molecular and Cytogenetic Abnormalities	Frequency in Childhood AML	References
Favorable	*CEBPA* gene single or double mutations	4–9%	[12]
	t(15;17)(q24;q21); *PML*::*RARA*	5–10%	[12,13]
	t(8;21)(q22;q22); *RUNX1*::*RUNX1T1*	15%	
	inv(16)(p13q22) or t(16;16)(p13;q22); *CBFB*::*MYH11*	10–15%	
	*NPM1*	4%	[12]
	t(16;21)(q24;q22); *RUNX1*::*CBFA2T3*	0.2%	[13,14]
Intermediate	del(7q)	3%	[13]
	t(9;11)(p22;q23); *KMT2A*::*AF9(MLLT3)*	6–9%	
	t(11;19)(q23;p13.1); *KMT2A*::*ELL*	1–2%	
	t(11;19)(q23;p13.3); *KMT2A*::*ENL(MLLT1)*	1%	
	t(10;11)(p12;q14); *PICALM*::*MLLT10*	<1%	
	t(3;5)(q25;q35); *NPM1*::*MLF1*	<0.5%	
	t(8;16)(p11;p13); *KAT6A*::*CREBBP*	<1%	
	t(1;22)(p13;q13); *RBM15*::*MKL1*	0.3%	
Adverse	Complex karyotype	8–17%	[13,15]
	Monosomy 5, del(5q)	1.2%	[13]
	Monosomy 7	3%	
	*FLT3*-ITD	10–20%	[16]
	t(10;11)(p12;q23) or ins(10;11) (p12;q23q13); *KMT2A*::*AF10(MLLT10)*	2–3%	[13]
	t(6;11)(q27;q23); *KMT2A*::*AF6(MLLT4)*	1–2%	
	t(4;11)(q21;q23); *KMT2A*::*AFF1*	-	[17]
	t(9;11)(p21;q23) *KMT2A*::*MLLT3*	-	
	t(5;11)(q35;p15); *NUP98*::*NSD1*	3–4%	[13]
	t(11;12)(p15;p13) *NUP98*::*KMD5A*	1–2%	
	t(7;12)(q36;p13); ETV6, MNX1	1%	
	inv(3)(q21q26.2) or t(3;3)(q21;q26.2); *GATA2*, *EVI1*(*MECOM*)	2%	
	t(6;9)(p22;q34); *DEK*::*NUP214*	<2%	[12,13]
	t(16;21)(p11;q22); *FUS*::*ERG*	0.4%	[13]
	inv(16)(p13q24); *CBFA2T3*::*GLIS2*	2–3%	
	t(9;22)(q34;q11); *BCR*::*ABL1*	0.6%	
Discussed	Monosomal karyotype	3–5%	
	Trisomy 8	10–14%	
	*FLT3*-TKD	7%	[16]
	*KIT* gene	<5%	
No significance	Hyperdiploidy (48~49–65 chr.)	11%	[13]
According to cryptic CA or to mutations	Normal karyotype	20–26%	

**Table 2:** Pediatric AML treatment outcomes based on therapeutic protocols.

Study Group	Study	Period	Patients	Probability of 3-Years/5-Years OS ± SD (%)	Probability of 3-Years/5-Years EFS ± SD (%)	Relapses (%)	Early Deaths	Deaths from Toxicities	Reference
AML-BFM	AML-BFM 2012 ^1^	2012–2018	164	82 ± 3/nd	69 ± 4/nd	22	*n* = 9	*n* = 6 3.7%	[14,17]
PPLLSG	AML-BFM 2012 ^1^	2015–2019	131	75 ± 5/nd	67 ± 5/nd	17	*n* = 8	*n* = 3 8%	[18]
COG	AAML1031 ^2^	2011–2016	1097	65.4 ± 3.1/nd	45.9 ± 3.2/nd	47.2 ± 3.2	nd	11.85 ± 5.2%	[19]
SJCGH	AML08 ^3^	2008–2017	285	74.8/nd	52.9/nd	21	*n* = 4	nd	[20]
DCOG	ANLL-97/ MRC AML-12 ^4^	1998–2002	118	nd/57 ± 5	nd/45 ± 5	45	*n* = 6	*n* = 1	[21]
	AML-15 ^5^	2002–2009	60	nd/61 ± 6	nd/49 ± 7	43	*n* = 4	*n* = 1	
	DB AML-01 ^6^	2009–2014	67	nd/72 ± 6	nd/48 ± 6	43	*n* = 2		

Abbreviations: OS—overall survival; SD—standard deviation; EFS—event-free survival; nd—no data; AML-BFM—Acute Myeloid Leukemia Berlin-Frankfurt-Münster studies; PPLLSG—Polish Pediatric Leukemia and Lymphoma Study Group; COG—Childhood Oncology Group; SJCRH—St. Jude Children’s Research Hospital; DCOG—Dutch Childhood Oncology Group. Study therapeutic target:^1^ improvement of event-free survival in pediatric AML comparing the use of clofarabine and etoposide in the 1stinduction course; ^2^ whether the addition of bortezomib to standard chemotherapy improves the survival in pediatric patients with newly diagnosed AML; ^3^ to identify effective and less toxic therapy for children with AML by introducing clofarabine into the first course of remission induction to reduce the exposure to daunorubicin and etoposide; ^4^ to optimize the treatment for younger patients with acute myeloid leukemia and high-risk myelodysplastic syndrome by comparing the induction options and the number of consolidation courses and whether consolidation should include transplantation; ^5^ to assess three combinations of drugs (cytarabine, daunorubicin and etoposide with daunorubicin and cytarabine and fludarabine, cytarabine, granulocyte colony-stimulating factor and idarubicin; Gemtuzumab ozogamicin was added to each combination) in consolidation and induction and ^6^ to improve survival in pediatric de novo AML.

## Data Availability

Not applicable.

## Abbreviations

ADCs	Antibody–drug conjugates
AE	adverse event
alloHSCT	allogeneic hematopoietic stem cell transplantation
AML	acute myeloid leukemia
AML-BFM	Acute Myeloid Leukemia Berlin-Frankfurt-Münster studies
ATAC	Assay for Transposase Accessible Chromatin
AZA	azacitidine
BCL-2	B-cell leukaemia/lymphoma 2
BiKEs	bispecific killer engager antibodies
BiTEs	bispecific T-engaging antibodies
CAR	chimeric antigen receptor
COG	Childhood Oncology Group
CR	complete remission
CRi	complete remission with incomplete blood count recovery
DAC	decitabine
DARTs	dual affinity retargets antibodies
DCOG	Dutch Childhood Oncology Group
DETs	differentially expressed protein-coding transcripts
DLT	dose-limiting toxicity
DNMT	DNA methyltransferase
EFS	event-free survival
FLT3-ITD	internal tandem duplication mutations in the juxtamembrane domain
FLT3-TKD	deletion in the tyrosine kinase domain
GO	Gemtuzumabozogamicin
HDACIs	histone deacetylase inhibitors
HDAC	Histone deacetylase
HLE	half-life extended
HMAs	hypomethylating agents
MRD	minimal residual disease
nd	no data
NIH	The National Institutes of Health
OS	overall survival
PDX	patient-derived xenografts
PPLLSG	Polish Pediatric Leukemia and Lymphoma Study Group
RFS	relapse-free survival
RTK	receptor tyrosine kinase
scFv	single-chain variable fragment
SD	standard deviation
SIRT	sirtuin protein family
SJCRH	St. Jude Children’s Research Hospital
TAA	tumor-associated antigen
Tregs	regulatory T cells
TriKEs	trispecific killer engager antibodies
TSG	tumor suppressor genes
WHO	World Health Organizatiοn

## Appendix A

**Table A1:** Food and Drug Administration (FDA) approval status of novel therapies for AML.

Drug	FDA Approval for Any Indication	Year of FDA Approval in AML
Midostaurin	Yes	2017
Gilteritinib	Yes	2018
Sorafenib	Yes	-
Venetoclax	Yes	2018
Azacitidine	Yes	2020
Decitabine	Yes	-
Panobinostat	Yes	-
Pracinostat	No	
Gemtuzumabozogamicin	Yes	2017
Camidanlumabtesirine	No	
CAR T cells	Yes	-
BiTEs	Yes	-

**Table A2:** Outcomes of the phase III clinical trials of novel methods of AML treatment.

Drug	Trial ^1^	Variable Used for Outcome Assessment					
		Median Overall Survival (Months)	Median Event-Free Survival (Months)	5-Year Event-Free Survival (%)	5-Year Relapse-Free Survival (%)	Complete Remission with Full or Partial Hematologic Recovery (%)	Median Relapse-Free Survival (Months)
Midostaurin	RATIFY	74.7 vs. 25.6 ^2^	8.2 vs. 3.0 ^2^	45 vs. 34	-	-	-
Sorafenib	SORAML	-	-	41 vs. 27 ^2^	53 vs. 36 ^2^	-	-
Gilteritinib	ADMIRAL	9.3 vs. 5.6 ^2^	2.8 vs. 0.7 ^2^	-	-	34 vs. 15.3 ^2^	-
Venetoclax	NCT03069352	8.4 vs. 4.1 ^2^	4.7 vs. 2 ^2^	-	-	48 vs. 13 ^2^	-
Azacitidine	QUAZAR	24.7 vs. 14.8 ^2^	-	-	-	-	10.2 vs. 4.8

^1^ All trials performed on the adult population; ^2^ vs. chemotherapy + placebo.
